# Synthesis, Spectroscopic Studies for Five New Mg (II), Fe (III), Cu (II), Zn (II) and Se (IV) Ceftriaxone Antibiotic Drug Complexes and Their Possible Hepatoprotective and Antioxidant Capacities

**DOI:** 10.3390/antibiotics11050547

**Published:** 2022-04-20

**Authors:** Samy M. El-Megharbel, Safa H. Qahl, Fatima S. Alaryani, Reham Z. Hamza

**Affiliations:** 1 Chemistry Department, Faculty of Science, Zagazig University, Zagazig 44519, Egypt; 2 Chemistry Department, College of Sciences, Taif University, Taif 21944, Saudi Arabia; 3 Biology Department, College of Sciences, Jeddah University, Jeddah 21959, Saudi Arabia; shqahal@uj.edu.sa (S.H.Q.); fsalaryani@uj.edu.sa (F.S.A.); 4 Biology Department, College of Sciences, Taif University, Taif 21944, Saudi Arabia; 5 Zoology Department, Faculty of Science, Zagazig University, Zagazig 44519, Egypt

**Keywords:** ceftriaxone, hepatotoxicity, metal complexes, oxidative stress

## Abstract

Magnesium, copper, zinc, iron and selenium complexes of ceftriaxone were prepared in a 1:1 ligand to metal ratio to investigate the ligational character of the antibiotic ceftriaxone drug (CFX). The complexes were found to have coordinated and hydrated water molecules, except for the Se (IV) complex, which had only hydrated water molecules. The modes of chelation were explained depending on IR, ^1^HNMR and UV–Vis spectroscopies. The electronic absorption spectra and the magnetic moment values indicated that Mg (II), Cu (II), Zn (II), Fe (III) and Se (VI) complexes form a six-coordinate shape with a distorted octahedral geometry. Ceftriaxone has four donation sites through nitrogen from NH_2_ amino, oxygen from triazine, β-lactam carbonyl and carboxylate with the molecular formulas [Mg(CFX)(H_2_O)_2_]·4H_2_O, [Cu(CFX)(H_2_O)_2_]·3H_2_O, [Fe(CFX)(H_2_O)(Cl)]·5H_2_O, [Zn(CFX)(H_2_O)_2_]·6H_2_O and [Se(CFX)(Cl)_2_]·4H_2_O and acts as a tetradentate ligand towards the five metal ions. The morphological surface and particle size of ceftriaxone metal complexes were determined using SEM, TEM and X-ray diffraction. The thermal behaviors of the complexes were studied by the TGA(DTG) technique. This study investigated the effect of CFX and CFX metal complexes on oxidative stress and severe tissue injury in the hepatic tissues of male rats. Fifty-six male rats were tested: the first group received normal saline (1 mg/kg), the second group received CFX orally at a dose of 180 mg/kg, and the other treated groups received other CFX metal complexes at the same dose as the CFX-treated group. For antibacterial activity, CFX/Zn complex was highly effective against *Streptococcus pneumoniae*, while CFX/Se was highly effective against *Staphylococcus aureus* and *Escherichia coli*. In conclusion, successive exposure to CFX elevated hepatic reactive oxygen species (ROS) levels and lipid peroxidation final marker (MDA) and decreased antioxidant enzyme levels. CFX metal complex administration prevented liver injury, mainly suppressing excessive ROS generation and enhancing antioxidant defense enzymes and in male rats.

## 1. Introduction

**Figure 1:**
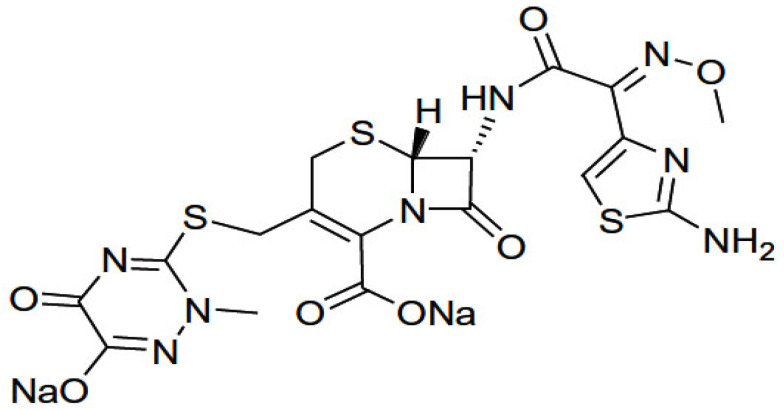
Chemical structure of sodium salt from the antibiotic ceftriaxone drug.

The antibiotic ceftriaxone drug (CFX) (Figure 1) is the third generation of antibiotic cephalosporin drugs. It is a parenteral cephalosporin that shows a high antibacterial activity [1]. This effect decreases urinary tract and respiratory infections, skin infections and skin structure, infections of bones and joints, pelvic inflammatory disease, non-enlarged gonorrhea, intra-abdominal infections and acute otitis media due to its surgical prophylaxis [2]. The drug shows high antibacterial activity and rare side effects, a long half-life of serum and is currently recommended for newborns that have Neisseria gonorrhea during childbirth [3]. CFX can be used as a stable mediator for acyl enzyme, preventing peptidoglycan cross-linking and thus disrupting the cell wall’s structural integrity [4].

CFX is also stable in relation to beta lactamases, which are formed using two bacterial types—Gram-positive and Gram-negative—and so can be used in the treatment of neonates [5]. Cefotaxime complexes with the general formula of MLCl (where L = cefotaxime drug; M = manganese (II)^+^, cobalt (II), nickel (II), cobber (II) and cadmium (II)) were prepared, and the ratio of metal to cefotaxime was 1:2, where cefotaxime was chelated via atoms of oxygen and nitrogen from groups of carboxylates, beta-lactam and aminothiazole. The antimicrobial activity of Cu (II) complexes is greater than free cefotaxime ligand [6].

CFX is an antibiotic that is commonly used for the treatment of bacterial infections such as abdominal and joint infection, skin and pelvic inflammatory diseases and bone and middle ear infection [7]. CFX vials are among the most prevalent types of antibiotics [8]. However, CFX produces a great deal of side effects, such as elevated liver enzymes and urea levels and diarrhea, and sometimes it induces thrombocytosis. Given the side effects of using this antibiotic drug in today’s health care system, it is essential to develop new drug complexes to elevate its wide activity and reduce any possible side effects [7].

Recently, some studies revealed that a novel nano-formula of the CFX drug had higher antibacterial activity against *E. coli* Gram-negative bacteria compared to the CFX drug alone. The greater antibacterial effect of the CFX nano-formula at a lower dose is another important finding with regard to the reduction of the antibiotic dose and to the cost-effective treatment of resistant microbes [9].

CFX complexes of Mn (II), Co (II), Cu (II), and Cd (II) were prepared in a molar ratio of 1:1 (M: CFX) and acted as pentadentate chelator with metal ions [10]. The antimicrobial activity of cadmium (II) complexes is more than free ceftriaxone ligand, while other complexes have almost the same effect as ceftriaxone. Ceftriaxone complexes of Fe (III), Co (II), Ni (II) and Cu (II) were prepared with octahedral geometry and molar ratios of 1:3 (CFX:M) [11]. Cefixime complexes with Mn (II), Co (II), Ni (II) and Cd (II) were prepared with a 1:1 molar ratio [12,13]. In addition, Fe (III) ceftriaxone complex was prepared with an octahedral geometry and was found to have high activity against bacterial species such as *Pseudomonas aeruginosa* [14].

Recently, there has been a great correlation between SARS-CoV-2 severity and hepatotoxicity especially induced by antibiotics. The severity of COVID-19 may be related with the risk of liver injury development [15]. There is increasing evidence that indicates that hepatotoxicity has been associated with the use of some medications in the treatment of patients infected with SARS-CoV-2 during the COVID-19 pandemic [15]. Recent epidemiological studies indicate different degrees of elevated liver hepatic enzymes with an incidence of 24.4%, particularly in liver transaminases, AST and ALT in COVID-19 patients. Liver injury associated with COVID-19 is defined as any damage that occurred in about 20–46.9% of COVID-19 patients to the liver due to either the treatment or pathogenesis of COVID-19 [16].

It has now been concluded that the severity of COVID-19 is correlated with the risk of liver injury. Additionally, it has been suggested that SARS-CoV-2 is greatly associated with liver injury and infection, which is still a matter of debate [17]. Meanwhile, in most cases, some treatments with antibiotics can cause liver damage during infection and can potentially cause some adverse effects, from severe bleeding to liver failure and even death. Hence, it is essential to find out novel antibiotic drug complexes with high antioxidant efficacy and low hepatic dysfunction to prevent such adverse effects on the hepatic tissues in COVID-19 patients, which is of great importance to alleviate pandemic diseases and enhance antioxidant defense systems [18].

A CFX complex of lead (II) was prepared and characterized, and the antibacterial activity (Gram-positive and Gram-negative bacteria) was evaluated [19]. Five CFX complexes were prepared with Ca (II), Zn (II), Fe (III), Au (III) and Pd (II) [20].

CFX metal complexes of Ca (II), Zn (II), Fe (III), Au (III) and Pd (II) metal ions were prepared, and all chemical characterizations were performed. Calcium (II), zinc (II), and iron (III) complexes have a distorted octahedral geometry, while Au (III) and Pd (II) are in the four-coordinate mode. The CFX ligand acts as a tetradentate towards the five metal ions through N (NH_2_) and O (triazine, β-lactam carbonyl, and COO groups). The assessment of the cytotoxicity of the Au (III) complex against HCT-116 and HepG-2, known as colon and hepatocellular carcinoma cells, showed that the IC_50_ of CFX/Au against HepG-2 cell line is 8.53 μg higher than that of HCT-116 cell line, at 20.5 μg [20].

Reactions of CFX with transition metal (II) ions with the general formula of [M(CFX)] (M = Mn, Co, Cu and Cd) and [Fe (CFX)Cl] were characterized using physicochemical and spectroscopic methods, where ceftriaxone acted as a dianionic pentadentate chelating agent through N_2_O_3_. The antibacterial activity was screened against several bacteria [21].

A CFX/Ca (II) complex was prepared and characterized [22] using elemental, TGA, IR spectroscopy and density functional theory calculations. The antibacterial and luminescence of ceftriaxone and the calcium complex were studied. The Ca (II) complex has a crystalline form. Cell parameters of the compound were determined. Ceftriaxone was chelated with calcium ion through oxygen (triazine cycle, lactam carbonyl and carboxylate groups) and nitrogen from the amino group of the thiazole ring. The antibacterial activity of the Ca (II) complex was screened against *Staphylococcus aureus, Escherichia coli* and *Pseudomonas aeruginosa*, and the results were compared with the activity of ceftriaxone disodium salt.

## 2. Experimental

### 2.1. Chemicals

All chemicals used were pure, and no further purifications were performed. Sodium salt of ceftriaxone ligand (Figure 1), MgCl_2_, CuCl_2_, FeCl_3_·6H_2_O, ZnCl_2_ and SeCl_4_ were from Sigma-Aldrich Chemical Company, Saint Louis, MO, USA.

### 2.2. Synthesis

CFX complexes were prepared by adding 1.0 mmol of MgCl_2_, CuCl_2_, FeCl_3_·6H_2_O, ZnCl_2_ and SeCl_4_ in CH_3_OH (40 mL solvent) with sodium salt to ceftriaxone (1.0 mmol) in CH_3_OH (40 mL). Then, refluxing was performed for about 4 h until colored precipitates were produced. After that, cooling, filtration for the solid complexity and washing using with hot methanol were performed; finally, the complexes were dried in a desiccator using dry CaCl_2_. All synthesized complexes were fully characterized as shown in (Table 1).

### 2.3. Experimental Animals

Fifty-six two-month-old male rats were used in this study. They were housed under standard conditions of temperature and supplied food ad libitum, and the study was ethically approved following all the international ethics guidelines for animal care. The treated groups were then divided into seven treated groups (eight rats in each group): Group 1 received 1 mL/kg saline solution (control group); Group 2 received CFX (180 mg/Kg) [23] orally for 30 consecutive days; Group 3, 4, 5, 6 and 7 received 180 mg/kg of Mg (II), Fe (III), Cu (II), Zn (II) and Se (IV) dissolved in saline solution for the same period of time.

Blood samples were collected, and serum samples were obtained after centrifugation at 10,000 rpm for approximately 25 min for biochemical tests. The male rats were gently dissected after light anesthesia by xylene/ketamine, and hepatic tissues were collected. Tissue samples were fixed in approximately 6% of neutral buffered formalin for the examination of histopathological sections.

### 2.4. Hepatic Functions and Antioxidant Assay

ALT and AST were evaluated in serum using a kit (Spinreact, Sant Esteve de Bas, Spain). Malondialdehyde, a final lipid peroxidation marker (MDA), was assayed in the hepatic tissues [24]. Superoxide dismutase (SOD) [25] and catalase (CAT) antioxidant enzymes were assessed in the homogenates of the liver tissues [26].

### 2.5. Histopathological Study

Liver tissue pieces were fixed in 6% neutral buffered formalin for 48 h, further processed for examination by hematoxylin and eosin (H&E) staining [27] and examined under a microscope (Leica Microsystems, New York, NY, USA).

### 2.6. Antibacterial Activities of CFX and Its Metal Complexes

The antimicrobial activity of the tested samples was determined by a modification of the Kirby–Bauer disc diffusion method. Antibacterial activity was tested in triplicate, and then the mean was calculated. In brief, 100 μL of the best bacteria was grown in 10 mL of fresh media until reaching an amount of approximately 108 cells/mL [28]. Then, 100 μL of the microbial suspension was spread into agar plates corresponding to the broth in which they were maintained. Isolated colonies of each organism that may play a pathogenic role were selected from the primary agar plates and tested for susceptibility by the disc diffusion method [29]. The Gram-positive bacteria *Bacillus subtilis* (Ehrenberg 23857™), *Streptococcus pneumonia* (Klein) Chester (6303™) and *Staphylococcus aureus* (23235™) and the Gram-negative bacteria *Escherichia coli* (BAA-2471™) and *Pseudomonas aeruginosa* (BAA-1744™) were incubated at 35–37 °C for 24–48 h. Afterwards, the inhibition zones’ diameters were measured in millimeters [30]. Standard discs of tetracycline drug served as positive controls for the antimicrobial activity, and a filter disc impregnated with 10 μL solvent (dist. H_2_O, DMSO) was used as a negative control.

The agar used was the Mueller–Hinton agar, which was tested continuously in terms of its pH. Furthermore, the depth of the agar in the plates was considered in the disc diffusion method [30].

### 2.7. Statistical Analysis

The results were presented as mean ± standard error. Comparisons within groups were conducted with a one-way ANOVA followed by post hoc analysis using SPSS version 17 (IBM^®^ SPSS^®^, Armonk, NY, USA).

## 3. Results and Discussions

### 3.1. Microanalytical and Conductance Measurements

Equal molar ratios for the metal ions (MgCl_2_, CuCl_2_, FeCl_3_·6H_2_O, ZnCl_2_ and SeCl_4_) and ligand ceftriaxone sodium salt (Na_2_CFX) produced colored metal complexes. C, H and N analysis data, magnetic susceptibility values and molar conductance (Λm = 15–25 Ω^−1^·cm^2^·mol^−1^) for ceftriaxone metal complexes are in Table 2. White, black, brown, white and yellowish white colors of the Mg (II), Cu (II), Fe (III), Zn (II)and Se (VI) complexes were shown, respectively. The data of the conductivity measurements prove the non-electrolytic character of Mg (II), Cu (II), Fe (III), Zn (II) and selenium (IV) complexes. Hence, CFX metal complex structures can be written as [Mg(CFX)(H_2_O)_2_]·4H_2_O, [Cu(CFX)(H_2_O)_2_]·3H_2_O, [Fe(CFX)(H_2_O)(Cl)]·5H_2_O, [Zn(CFX)(H_2_O)_2_]·6H_2_O and [Se(CFX)Cl_2_]·4H_2_O. The ceftriaxone complexes are insoluble in most organic and inorganic solvents, such as H_2_O, CH_3_OH, C_2_H_5_OH, CHCl_3_, CH_2_Cl_2_ and CCl_4_, but they are soluble in DMSO and DMF. The contents of metal were measured gravimetrically [31]. The produced complexes were elucidated using different tools of analysis such as C, H and N, molar conductance, IR, ^1^HNMR, electronic spectra, magnetic, SEM, TEM and XRD analyses.

### 3.2. FTIR Spectral Studies

**Figure 2:**
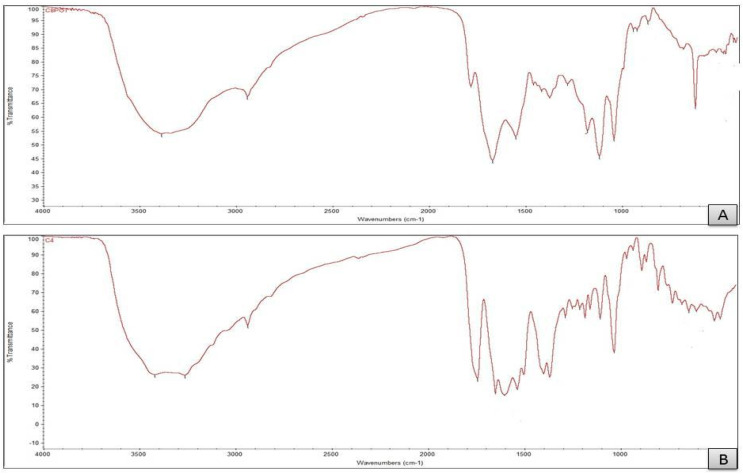

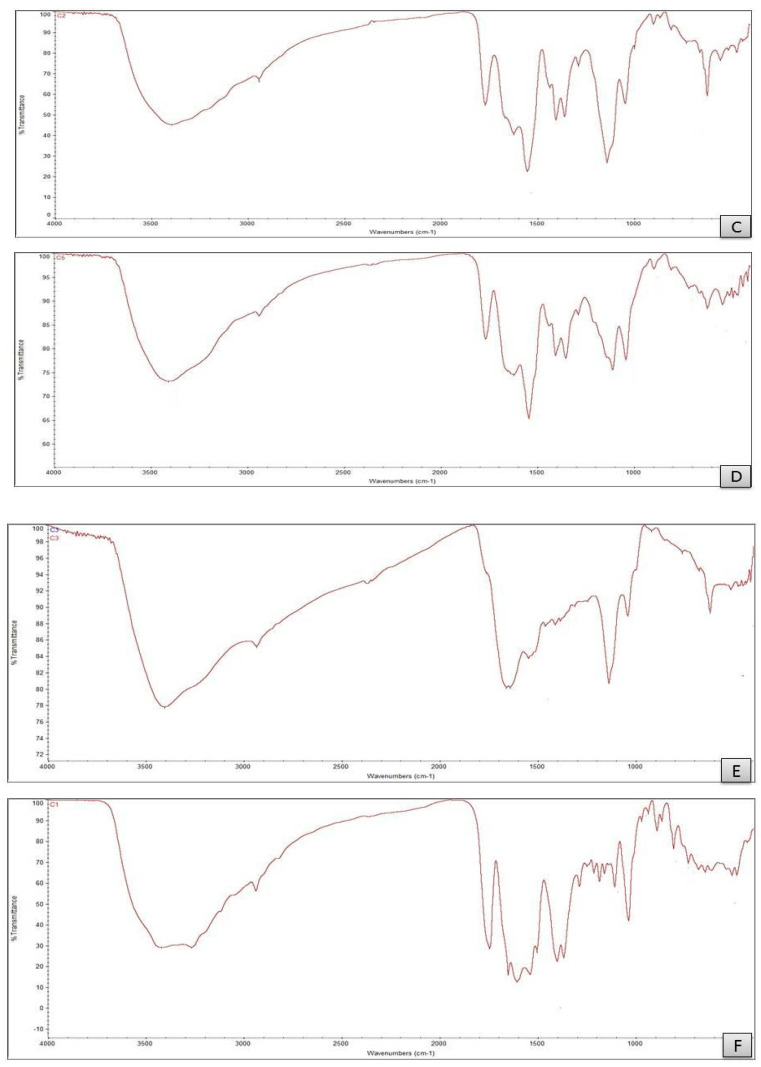
FT-IR of (**A**) CFX, (**B**) CFX/Mg, (**C**) CFX/Cu, (**D**) CFX/Fe, (**E**) CFX/Se and (**F**) CFX/Zn.

Infrared spectroscopy is an essential tool for identifying the main functional groups of organic molecules. The CFX free ligand has more than one donor atom, such as the O atom from the thiazole cycle, N atom of the NH_2_ group and atoms of O from carboxylate, lactam and amide carbonyl groups. The mode of chelation for free ceftriaxone drug ligand towards metal ions Mg (II), Cu (II), Fe (III), Zn (II) and Se (VI) was studied. The IR for ceftriaxone and its metal complex are similar and are recorded in Table 3 and Figure 2. Generally, the absorption frequency for carbonyl ring groups for free ceftriaxone ligands will be shifted to lower wave numbers after complexation.

After the reaction of CFX with metal ions, there are shifts in the stretching vibrations of ν(C=O) βlactam and ν(C=O) triazine to 1766–1621 cm^−1^ and 1552–1536 cm^−1^, respectively [32]. These shifts can be attributed to the contribution of oxygen atoms to the chelation with metal ions. The frequencies of symmetric stretching for the carboxylate group vs. (COO^−^) shift to 1399–1309 cm^−1^ [10,28]. Based on frequencies of the FTIR spectra of Na_2_CFX and its metal complexity, a shift in the band appeared at 3410 cm^−1^ assigned to a stretching vibration ν(N–H) of the amino group to wavenumbers 3380–3395 cm^−1^, confirming the participation of N atoms of the NH_2_ group in the coordination with metal ions. For the monodentate coordination of the COO- group, according to the explanations of Deacon and Phillips [31,32], a difference larger than >200 cm^−1^ disproves this, whereas one smaller than 200 cm^−1^ indicates that coordination is monodentate. These shifts confirm the involvement of the oxygen from the (COO)carboxylate group, the oxygen from the (C=O) carbonyl group of β-lactam, nitrogen from the amine group and the oxo group of the triazine ring in the coordination. All these data are in agreement with previous studies showing a tetradentate behavior of ceftriaxone ligand [10,31]. The spectral band, which is broad in all ctx complexes that appear at 3264–3290 cm^−1^, is due to the ν(OH) of hydrated and coordinated water molecules [33]. There are new bands that appear at the range of 513–461 cm^−1^ corresponding to stretching vibration bands ν(M–N) for the metal complexes (with no free ligand), confirming that the -NH_2_ group of the thiazole moiety is chelated with metal ions. The chelation of the group of -NH_2_ with metal ions is not the only explanation for these absorption bands. For CONH and C=N-OCH_3_ groups, nitrogen atoms could react with metal ions in solid complexes; however, coordination through nitrogen atoms and COO and lactam CO groups is prevented due to steric constraints. In addition, the stretching vibration for CONH moiety and C=N of C=N-OCH_3_ appeared in free ligands of ceftriaxone at 1178 and 1551 cm^−1^, respectively, and did not shift for all ceftriaxone metal chelates, confirming that these groups did not participate in coordination. The new bands appear in the range of 541–678 cm^−1^ for ceftriaxone complexes and are absent for free ceftriaxone; these are assigned to stretching vibrations of ν(M–O). At the range of 1700–1600 cm^−1^ in ceftriaxone metal complexesm there are broad bands that have high intensity and low resolution regarding the overlap between several vibrational modes, such as ν(C=O)-amide, ν(C=O)-triazine, ν_as_(COO^–^), ν(C=C) and ν(C=N). This is in an agreement with previous data for polydentate ceftriaxone ligands [34,35].

### 3.3. Electronic Spectra

**Figure 3:**
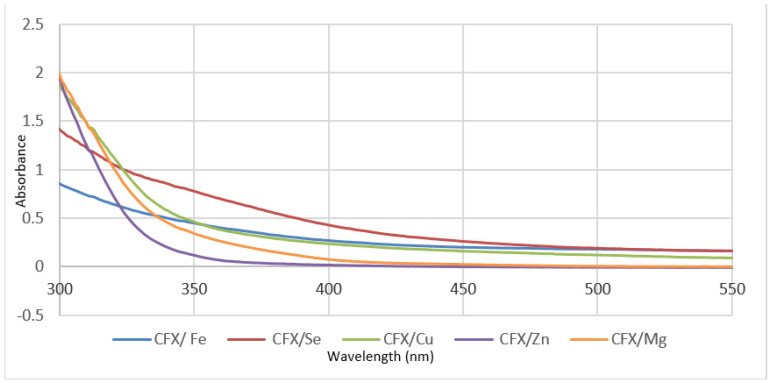
U.v.-vis. spectra of CFX metal complexes.

The u.v.-vis. spectra for sodium salt of the CFX free ligand and its metal complexes measured within 200–800 nm using DMSO as a solvent are shown in Figure 3.

The u.v.-vis. spectra for ceftriaxone and its complexes give an absorption maximum at 250–270 nm assigned to the π→π* transition due to orbital molecular energy levels of the nitrogen–carbon–sulfur moiety [36] at 285–300 nm, due to transitions of the π→π* band of intraligands in moieties of triazole and 1,3-thiazole. The appearance of bands in the region of 350–390 nm is related to the n→π* type of transition in intraligands, and this is in agreement with literature data for sulfur atom transitions [37]. The bands related to sulfur atoms are not shifted, confirming that S atoms do not participate in chelation with metal ions. The Fe(III) complex gives very weak absorption bands, and this may be attributed to spin-orbit forbidden transitions. The selenium(VI) complex gives a weak band at around 500 nm, while the Cu(II) complex exhibits a transition of d–d, which appears as a weak band around at 400 nm, suggesting that copper(II) and Se(IV) complexes form six coordinate chelates [37]. The difference in wavelength values in CFX complexes is more than that for free ceftriaxone ligand, confirming the participation of Mg (II), Cu (II), iron (III), Zn(II) and Se (VI) with CFX complexes [38].

### 3.4. Magnetic Measurements

The value of magnetic moment μeff for the Fe (III) complex is 5.92 B.M., which is consistent with d^5^ high spin systems with five electrons unpaired. This value of effective magnetic moments is located within the high spin octahedral geometry. The magnetic moment value for copper(II) ceftriaxone complex at room temperature is 2.31 B.M., confirming that Cu metal ions are present in an excess amount inside the chelation sphere. The lowered values for magnetic moments are related to antiferromagnetic interactions between the ions, while the higher values for magnetic moments show that ferromagnetic interactions rarely occurred. The value of magnetic moment μeff for the Se (VI) complex is 5.98 B.M.—this value of effective magnetic moments is located within the high spin octahedral geometry.

### 3.5. ^1^H-NMR Study

For free ceftriaxone Na_2_CFX, the ^1^HNMR spectrum data obtained can be summarized as follows.

**Figure 4:**
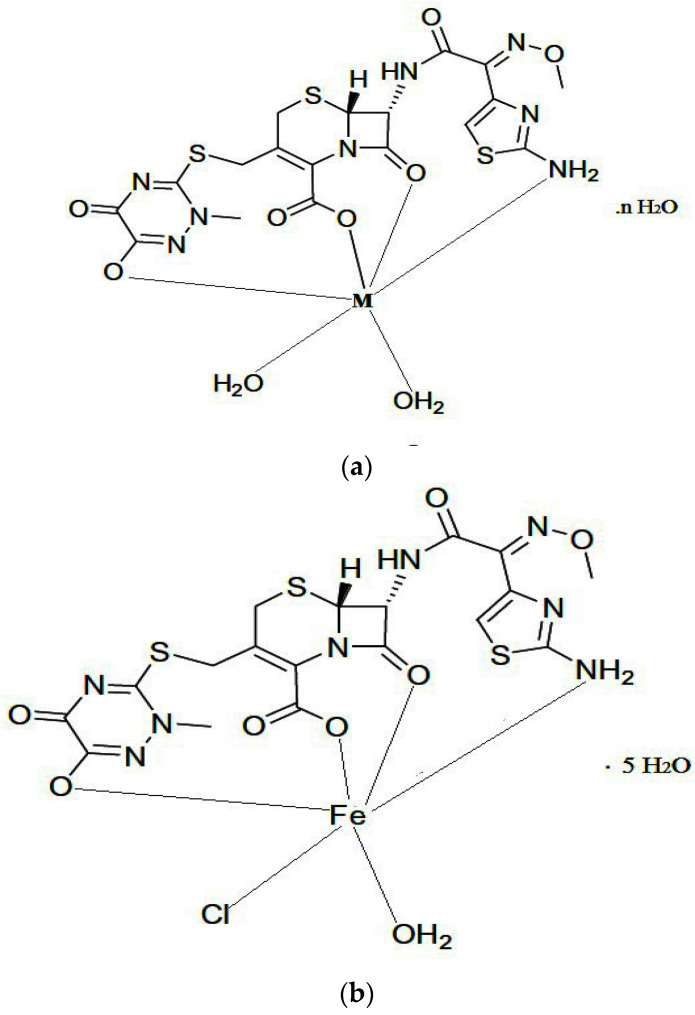

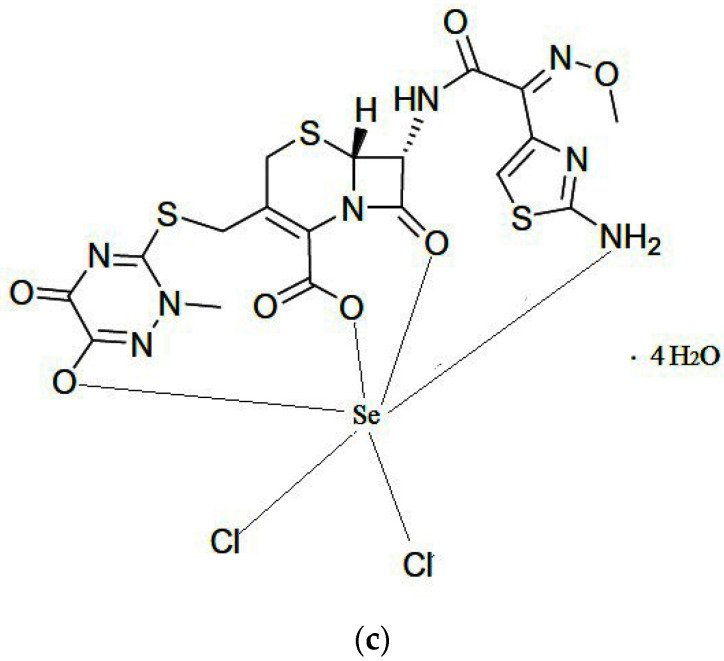
The structure of the prepared CFX complexes. (**a**) M = Mg, Cu and Zn/ CFX, n = 4,3 and 6 respectively. (**b**) Fe/CFX complex. (**c**) Se /CFX complex.

At 3.368 [CH_2_ of thiazine, 2H] at δ 3.489, [N-CH_3_ of triazine, 3H] at 3.889, [=N-O-CH_3_, 3H] at 3.960 [S-methylen, 2H], at 5.069 [β-lactam, 1H] and 6.910 [thiazol ring, 1H]. The spectra of proton nuclear magnetic resonance for [Mg(CFX)(H_2_O)_2_]·4H_2_O, [Zn(CFX) (H_2_O)_2_]·6H_2_O and [Se(CFX)Cl_2_]·4H_2_O complexes in Table 4 show occurrences of upward shifts of the field in the reading signals for Na_2_CFX, confirming the coordination between Mg (II), Zn (II) and Se (VI) metal ions and ceftriaxone ligand. Based on C, H and N, molar conductance, IR, ^1^HNMR, electronic spectra and magnetic analyses, the suggested structures for ceftriaxone complexes are shown in Figure 4.

### 3.6. XRD Analysis

**Figure 5:**
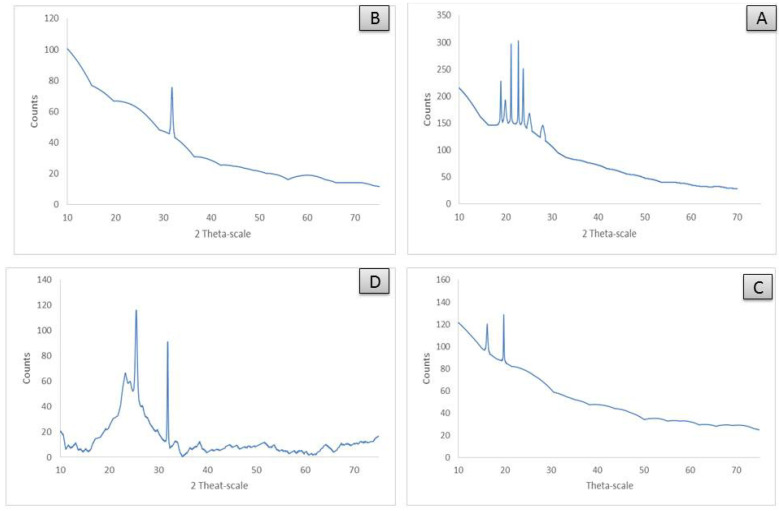
XRD for (**A**): [Zn(CFX)(H_2_O)_2_]·6H_2_O; (**B**): [Cu(CFX)(H_2_O)_2_]·3H_2_O; (**C**): [Fe(CFX)(H_2_O)_2_]·5H_2_O; (**D**): [Se(CFX)Cl_2_]·4H_2_O.

XRD analysis is an essential technique for identifying the nature of crystallinity in metal complexes. The patterns of X-ray diffraction powder for [Zn(CFX)(H_2_O)_2_]·6H_2_O, [Cu(CFX)(H_2_O)_2_]·3H_2_O, [Fe(CFX)(H_2_O)_2_]·5H_2_O and [Se(CFX)Cl_2_]·4H_2_O complexes were characterized at room temperature using X-ray diffraction by Cu Kα radiation and are shown in Table 5. The X-ray diffraction patterns for Zn (II), Cu (II), Fe (III) and Se (VI) CFX complexes were measured in the range of 2θ = 10–70° and are shown in Figure 5, and they have an amorphous behavior with a nano-form structure, as shown in Figure 5. Depending on the Scherrer relationship, the sizes of particles were detected [39] with the help of the full width at half maximum (FWHM) and have a 15–57 nm range.

### 3.7. SEM and TEM Investigations

**Figure 6:**
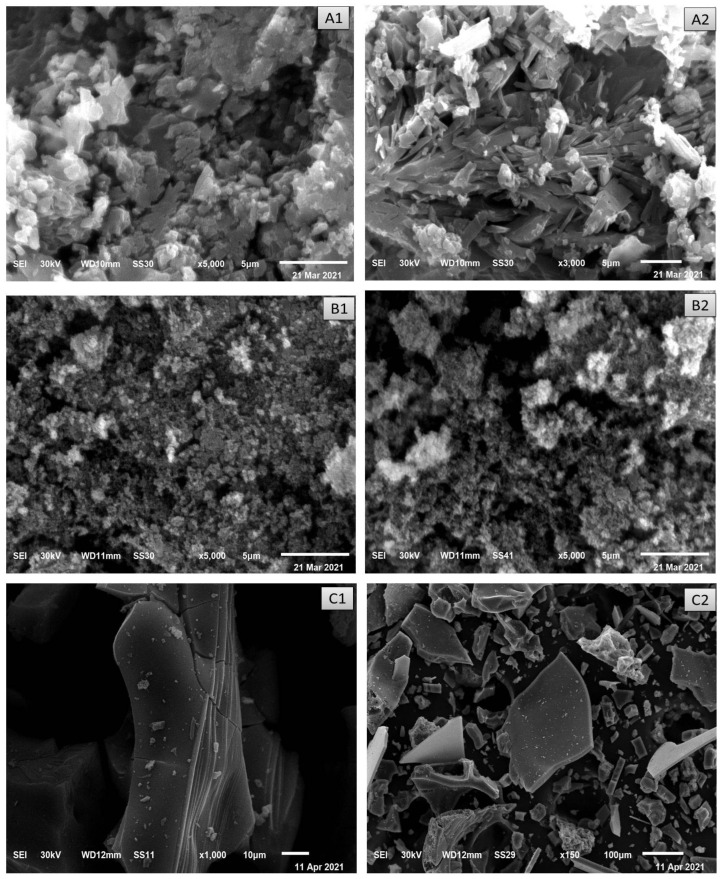

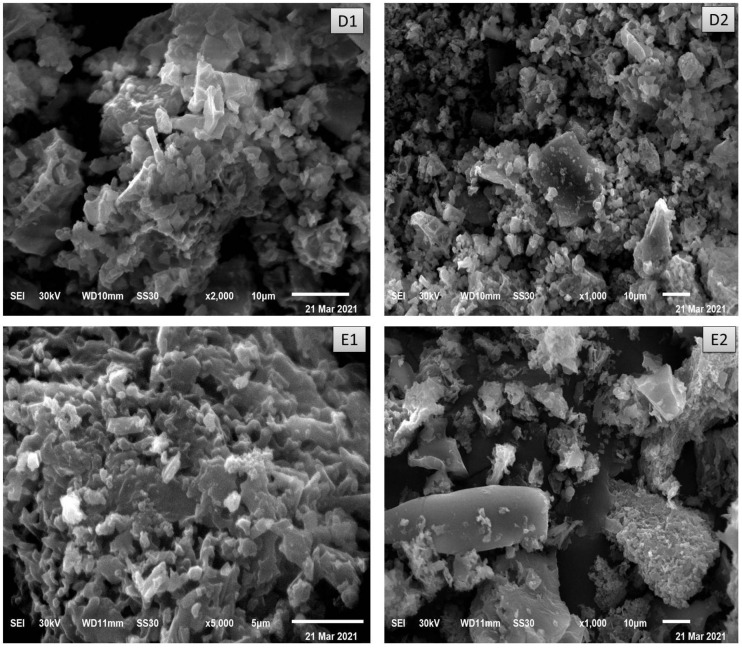
SEM (**A1**,**A2**) Mg, (**B1**,**B2**) Cu, (**C1**,**C2**) Fe, (**D1**,**D2**) Se and (**E1**,**E2**) Zn CFX complexes.

TEM and SEM tools are essential techniques for identifying the surface morphology of synthesized metal complexes. Images of SEM for ceftriaxone metal complexes are shown in Figure 6. SEM images show homogeneity and uniform aggregation of Mg (II), Cu (II), Fe (III), Zn (II) and Se (VI) CFX complexes.

**Figure 7:**
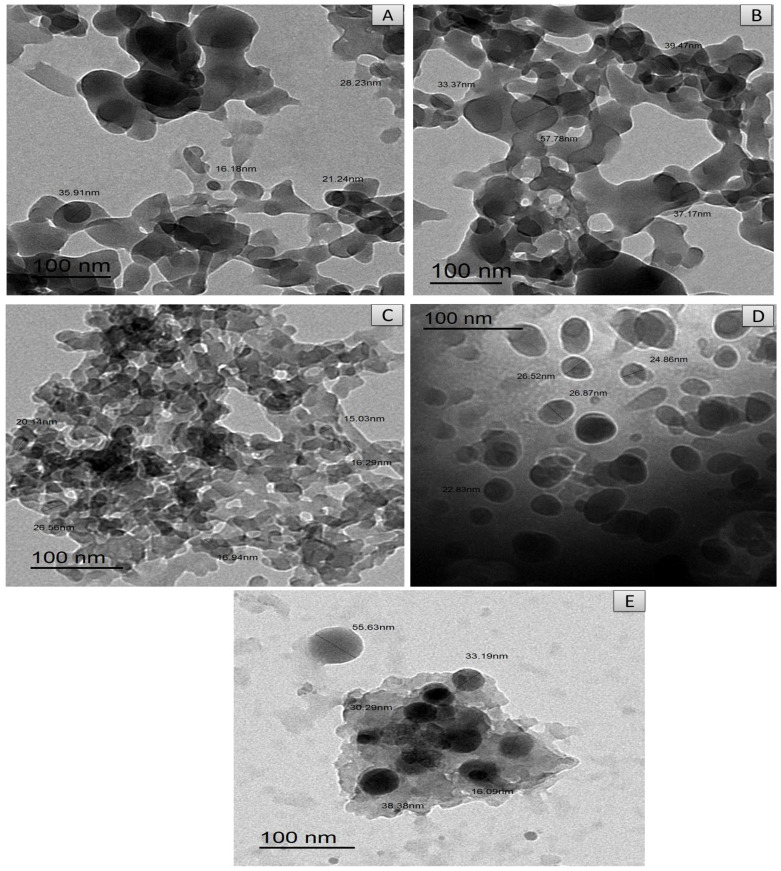
TEM (**A**) Cu, (**B**) Mg, (**C**) Fe, (**D**) Zn and (**E**) Se CFX complexes.

Images of TEM for [Mg(CFX)(H_2_O)_2_]·4H_2_O, [Cu(CFX)(H_2_O)_2_]·3H_2_O, [Fe(CFX)(H_2_O)_2_]·5H_2_O, [Zn(CFX)(H_2_O)_2_]·4H_2_O and [Se(CFX)Cl_2_]·4H_2_O complexes are shown in Figure 7 and refer to the formation of spherical black spots with nanoparticles in the ranges of 33–57 nm, 16.18–35.91 nm, 15.03–26.87 nm, 22.83–26.87 nm and 16.09–55.63 nm, respectively. The nano-sized CFX metal complexes were observed with TEM to have a particle size of 15.03–57 nm, which is in agreement with XRD data.

### 3.8. Thermal Analysis

Thermal analysis is an essential tool used for the characterization of metal complexes where, when heating a compound, its weight loss increases. In addition, DTG differential thermogravimetric analysis (DTGA) is used to study the thermal stability of compounds and the weight loss at different temperatures. TGA confirmed the successful chelation of CFX with different metal ions. Thermogravimetric analysis (TGA) and differential (DTGA) analysis for Mg (II), Cu (II), Fe (III), Zn (II) and Se (IV) CFX complexes were carried out in a temperature range of 30–800 °C under an N_2_ atmosphere. The first decomposition endothermic step occurred in a temperature range 30–130 °C and corresponded to a loss of molecules of crystalline water. The second cracking step was carried out between 130–380 °C owing to a loss of ceftriaxone ligand. The third stage of decomposition occurred in temperature ranges of 380–530 °C and 530–620 °C, representing the evolution of NH_3_, CO_2_ and HNCO. The final residual products for all ceftriaxone metal complexes are metal oxides.

### 3.9. Ceftriaxone Metal Complexes Alleviate Hepatic Injury in Male Rats

Excessive CFX exposure for 30 consecutive days afforded a significant increment in serum activities of ALT and AST in male rats. However, in contrast, the supplementation of CFX complexes induced significant improvements in liver enzymes, and the best ameliorations were recorded in CFX/Mg, CFX/Zn and CFX/Se, respectively, as compared to the CFX treated group alone, as shown in Table 5.

### 3.10. Ceftriaxone Metal Complexes Alleviate Oxidative Injury in the Hepatic Tissues and Structural Alterations of Male Rats Exposed to Ceftriaxone

Reactive oxygen species levels manifested a marked elevation in the hepatic tissues of CFX-exposed male rats. Supplementation of CFX metal complexes reduced the excessive hepatic generation of free radicals, as recorded in the CFX-treated group. Hepatic MDA levels were significantly elevated in the CFX-treated group only. These changes were mainly inverted in the group to which CFX metal complexes were administered, especially CFX/Zn and CFX/Mg, respectively.

On the contrary, male rats exposed to CFX exhibited a high decline in hepatic antioxidant enzyme (GSH, SOD and CAT) activities. GSH, SOD activity and CAT declined markedly as compared with the control group. Oral supplementation of CFX metal complexes elevated the hepatic antioxidant enzymes as compared to CFX alone, especially for CFX/Mg, CFX/Zn and CFX/se, CFX/Cu and CFX/Fe, in the same order as shown in Table 6.

**Figure 8:**
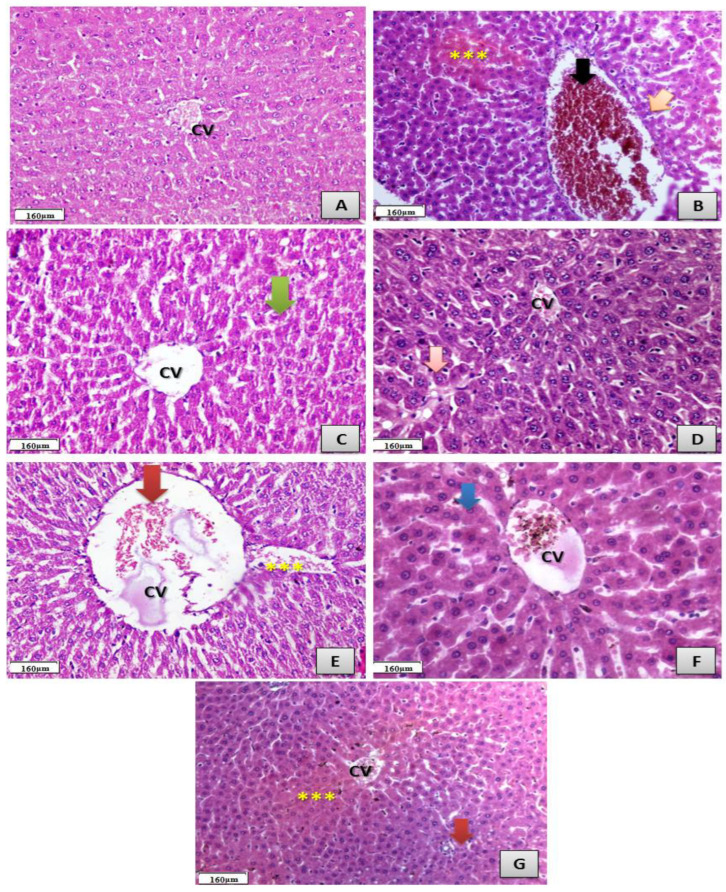
Photomicrographs showing histological sections of the hepatic tissues. (**A**) Control group showing normal hepatic structure and normal central vein (CV) (H&EX400). (**B**) CFX-treated group showing severe toxicity in the form of the hypertrophy of hepatocytes (orange arrow) with the appearance of binucleated hepatocytes and increased eosinophilia, granular cytoplasm and vesicular nuclei (***); the central vein is dilated and filled with hemorrhage and necrotic tissue (black arrow), with focal necrosis in some hepatocytes with increased eosinophilia and nuclear disappearance and the accumulation of a few mononuclear inflammatory cells in blood sinusoids (H&EX400). (**C**) CFX/Mg-treated group showing an almost normal hepatic structure (green arrow) with a normal sized central vein (CV) (H&EX400). (**D**) CFX/Zn-treated group showing very mild toxicity in the form of the hypertrophy of hepatocytes with granular eosinophilic cytoplasm and vesicular nuclei and the appearance of some binucleated cells (orange arrow), with a mild congested central vein which contains (CV) mild brown particles of bilirubin and single hepatocyte necrosis (H&EX400). (**E**) CFX/Se-treated group showing an almost normal hepatic structure with normal hepatocytes with a moderately enlarged central vein (CV) filled with some red blood cells (red arrow) with some mild detachment (***) (H&EX400). (**F**) CFX/Cu-treated group showing an almost normal hepatic structure with ballooning cytoplasm (blue arrow) and a central vein (CV) filled with some red blood cells (H&EX400). (**G**) CFX/Fe-treated group showing mild toxicity in the form of the hypertrophy of hepatocytes with the. appearance of binucleated hepatocytes and increased eosinophilia, granular cytoplasm and vesicular nuclei (red arrow); the central vein is normal in diameter with mild congestion (CV) and ballooning degeneration in some hepatocytes (***), with increased eosinophilia around the central vein indicating the beginning of necrosis (H&EX400).

Histological sections of hepatic tissues of different treated groups as shown in (Figure 8) which showed marked hepatic alterations and some atrophy in CFX treated group only, while there was marked improvement recorded in hepatic tissues of other treated groups with either CFX/Mg, CFX/Zn, CFX/Se, CFX/Cu and CFX/Fe, Meanwhile the marked improvement was recorded in CFX/Mg, CFX/Zn and CFX/Cu treated groups respectively.

### 3.11. Antibacterial Activity Evaluation

**Figure 9:**
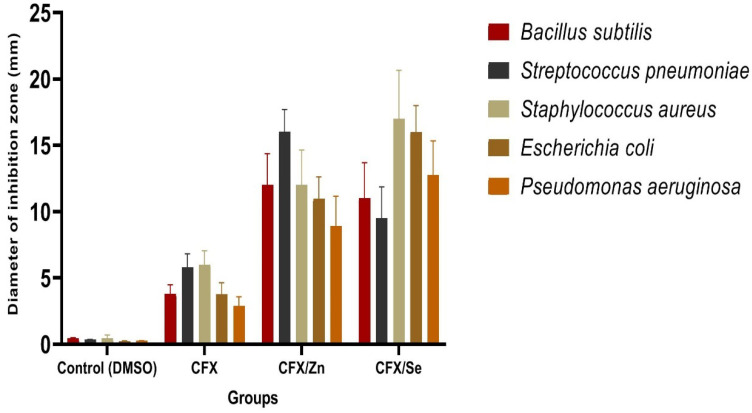
Antibacterial activity of CFX and Zn (II) and Se (IV) metal complexes.

Biological evaluations were performed for CFX complexes against Gram-positive (*Bacillus subtilis*, *Streptococcus pneumoniae* and *Staphylococcus aureus*) and Gram-negative (*Escherichia coli* and *Pseudomonas aeruginosa*) bacteria. Results from the agar disc diffusion tests for the antimicrobial activities are presented in Table 6 and demonstrated in Figure 9. The diameters of the zone of inhibition (in mm) of the standard drug Amikacin (Aminoglycoside) (C_22_H_43_N_5_O_13_) against Gram-positive bacteria *B. subtilis* and *S.aureus* and Gram-negative bacteria *E. coli* and *P. aeruginosa* were found to be 36, 30, 31 and 35 mm, respectively. Under identical conditions, Table 7 and Figure 9 show that all complexes were found to be efficient, with a high antimicrobial activity that exceeded CFX itself.

## 4. Discussion

A serious problem now facing humanity is oxidative damage and its dangerous consequences for human health and hepatic vitality. The world now needs powerful antibiotics with potent antioxidant activities and fewer side effects for the liver tissues to reinforce the immune system and fight against resistant microbes and microorganism. Thus, the current study was designed to synthesize novel metal complexes of ceftriaxone (CFX) with Mg (II), Fe (III), Cu (II), Zn (II) and Se (IV) to investigate the potency of the hepatoprotective effects and antioxidant capacities of CFX complexes and determine if they succeed in the elevation of antioxidant capacities to investigate novel compounds with high antioxidant capacities.

The oxidative injury of biomolecules is the major concern in the pathogenesis of a large number of diseases such as cancer, degenerative diseases, metabolic diseases and even dangerous instances of SARS-CoV-2. Thus, it is very important to investigate the role of potent novel metal complexes to prevent oxidative stress and the treatment of side effects and diseases induced by such oxidative stress.

Liver injury caused by drugs ranges from mild biochemical abnormalities to chronic liver failure. The majority of adverse liver reactions occur in most antibiotic treatments [23].

Some antibiotics are considered to be a common cause of liver injury. The hepatotoxicity that occurs is usually associated with hepatic impairment [40]. CFX is a broad-spectrum antibiotic with potent activity against Gram-positive and Gram-negative bacteria [41].

The hepatotoxicity caused by CFX appears after an average of 9–11 days [42,43]. Previous studies have reported high aspartate aminotransferase (ALT) and alanine aminotransferase (AST) activities with the administration of CFX [44,45], and this concept coincides with the current findings that confirmed that CFX causes hepatic damage as a result of elevations in some biochemical parameters such as AST, ALT and low-density lipoprotein (LDL) as well as a decline in high-density lipoprotein (HDL) concentrations [46,47,48].

Based on the previous background, researchers have demonstrated that CFX is widely used as a third-generation cephalosporin antibiotic that has a broad spectrum of bactericidal activity. However, previous evidence has indicated that CFX carries a risk of the elevation of liver enzymes, with liver injury as an adverse effect [48].

In agreement with previous findings [23] that reported that the oxidative stress mediated by oxygen free radicals (ROS) has been implicated as a common link between chronic liver damage and hepatic fibrosis, the current study demonstrated that the administration of CFX resulted in a significant decline in hepatic GSH. Conversely, a significant increment in the level of hepatic MDA (a marker of lipid peroxidation) was shown. The increase in MDA level was more pronounced in rats in CFX-treated groups.

The novel finding of our current study is the improvement effects of CFX metal complexes, especially in groups treated with CFX with Mg, Zn, Se and Cu complexes, respectively, as they improved hepatic function serum levels and ameliorated the hepatic structure greatly.

Our explanation depends on the functional activity of the used metals, as previous researchers demonstrated the importance of Se metal in enhancing the glutathione levels of hepatic tissues, leading to enhancements of the whole antioxidant defense system [49,50,51,52] and a decline in the excessive generation of reactive oxygen species.

Metal ions are required to keep the human body healthy because several critical biological functions in humans depend upon their presence, and their absence or scarcity may lead to diseases. Magnesium (Mg) belongs to the main group of elements and is required mainly for fat and carbohydrate metabolism. Meanwhile, zinc (Zn) belongs to the transition metal group of elements. Zn is a vital element due to its strong binding to proteins. Zn also is exceptionally stable with respect to oxidation and reduction, and thus it does not participate in redox reactions. Additionally, Zn^2+^ shows a strong preference in enzymes for a tetrahedral coordination over an octahedral one [53], and all these previous aspects confirm the findings of the current study and the great improvement activities of CFX metal complexes, especially with Zn^2+^, in the alleviation of the hepatic side effects induced by CFX alone.

A deficiency of Fe leads to anemia, as it is known that Fe is a part of hemoglobin; besides this, a deficiency of Cu leads to heart diseases and anemia. The importance of all of these metals and especially in conjugation with CFX has been proved chemically in alleviating oxidative stress and providing high hepatic protection. The Gram-negative strains are exposed to various stress conditions during pathogenesis, of which the stress of acids serves as a major defense mechanism in the host [54]. Such environments are encountered, and this supports the main hypothesis of the study and proved the hepatoprotective and antioxidant capacities of two synthesized CFX complexes with either Zn (II) or Se (IV); in addition, the two complexes showed antibacterial activity besides their abilities to reduce free radicals’ production.

## 5. Conclusions

The current study aimed to synthesize five ceftriaxone complexes by the reaction of ceftriaxone sodium salt with (Mg^2+^, Zn^2+^, Fe^3+^, Cu^2+^ and Se^4+^) ions. The structures of the CFX complexes have been explained using microanalytical, molar conductance, IR, ^1^HNMR, UV–Vis, magnetic, SEM, TEM and X-ray diffraction analyses. Mg (II), Cu (II), Zn (II), Fe (III) and Se (VI) complexes form six-coordinate systems with a distorted octahedral geometry. The obtained results clarified that ceftriaxone metal complexes, especially (CFX/Mg, CFX/Zn and CFX/Se), greatly ameliorated hepatic enzyme functions and enhanced the antioxidant activities of hepatic tissues while reducing the excessive triggering of excessive reactive oxygen species (ROS) and the protection of hepatic structures as compared with ceftriaxone-treated groups alone. For antibacterial activity, the CFX/Zn complex was highly effective against *Streptococcus pneumoniae*, while CFX/Se was highly effective against *Staphylococcus aureus* and *Escherichia coli*. These results are very promising in providing protection for the hepatic tissues and reducing damaging effects and the severe oxidative stress induced by antibiotics on liver tissues, especially during the COVID-19 pandemic; in addition, it brings the recent research that correlated the damage of hepatic functions and severe instances of SARS-CoV-2 on the health of the body up to date.

## Figures and Tables

**Table 1:** Instrumentations and experimental analyses.

Instrument	Measurement
Perkin Elmer CHN 2400 (USA)	Contents of C, H and N
Jenway 4010 conductivity meter	Electrolytic or non-electrolytic character
Bruker FTIR Spectrophotometer (4000–400 cm^−1^)	IR measurements
UV2 Unicam UV/Vis Spectrophotometer	Electronic spectra
varian mercury VX-300 NMR spectrometer	The ^1^H-NMR
Sherwood scientific magnetic balance using Gouy method	Magnetic measurements
Quanta FEG 250 equipment	Scanning electron microscopy (SEM) images
X’Pert PRO PAN analytical X-ray powder diffraction, target copper with secondary monochromate	X-ray diffraction patterns
JEOL 100s microscope	Transmission electron microscopy images (TEM)

**Table 2:** Elemental analysis and conductivity measurements for ceftriaxone complexes.

Complexes	M.Wt	Color	Elemental Analysis			Λm (Ω^−1^cm^2^mol^−1^)	Magnetic Moment (BM)
			C	H	N		
[Mg(CFX)(H_2_O)_2_]·4H_2_O C_18_H_28_N_8_O_13_S_3_ Mg	684.30	White	(31.56) 31.78	(4.09) 4.12	(16.36) 16.16	15	-
[Cu(CFX)(H_2_O)_2_]·3H_2_O C_18_H_26_N_8_O_11_S_3_Cu	705.546	black	(30.61) 30.58	(3.68) 3.94	(15.82) 15.59	17	2.31
[Fe(CFX)(H_2_O)(Cl)]·5H_2_O C_18_H_28_ClN_8_O_13_S_3_Fe	751.98	Greenish black	(28.72) 28.46	(3.72) 3.49	(14.89) 14.65	21	5.92
[Zn(CFX)(H_2_O)_2_]·6H_2_O C_18_H_30_N_8_O_14_S_3_Zn	762.09	White	(28.34) 28.61	(3.93) 3.94	(14.96) 14.32	16	-
[Se(CFX)Cl_2_]·4H_2_O C_18_H_26_Cl_2_N_8_O_12_S_3_Se	694.96	Yellowish white	(31.08) 31.37	(3.74) 384	(16.11) 16.57	25	5.98

**Table 3:** Infrared frequencies (cm^–^^1^) for ceftriaxone and its complexes.

Assignments		Compounds				
	Na_2_CFX	Mg (II)	Cu (II)	Fe (III)	Zn (II)	Se (VI)
ν(N–H)	3410	3385	3395	3390	3380	33,385
ν(O–H); H_2_O	-	3264	3280	3270	3265	3290
ν(C=O); lactam ring	1782	1744	1769 1670	1766 1657	1744 1649	1766 1660
ν_as_(C–N) + ν(C=O)_OCO_ ν(COO)	1604	1537 1503	1550	1543	1537 1503	1545
δ(CH_2_) + δ(CH_3_)	1416	1408	1403	1404	1400	1409
δ(CH)lactam + ν_as_(COO)	1374	1367	1359	1352	1366	1309
ν_s_(C–N)triazine	1281	1286	1288	1287	1266	1242
δ(CH)lactam + δ_w_(CH_3_)	1260	1250	1245	1232	1212	1242
δ_r_(CH_3_)	1178	1171	1138	1146	1106	1135
δ(CH)aminothiazol	1040	1033	1045	1040	1034	1039
ν(N–O)	921 864	889 805	898 864	895 805	890 805	918 760
ν(M–O)	-	645 606	657 620 552	618 541	678 645 610	636 619
ν(M–N)	-	510 492	510 487	485 461	507 482	513 475

**Table 4:** HNMR spectral assignments of ceftriaxone and its complexes.

Signals	Na_2_CFX Ligand	Mg (II)	Zn (II)	Se (VI)
[2H, CH_2_ of thiazine]	3.368	3.352	3.342	3.318
[3H, N-CH_3_ of triazine ring]	3.489	3.375	3.312	3.254
[3H, =N-O-CH_3_]	3.889	3.879	3.785	3.547
[2H, S-CH_2_]	3. 960	3.864	3.687	3.758
[1H, β-lactam]	5.069	4.652	4.758	4.989
[1H, of thiazol ring]	6.910	6.897	6.874	6.987

**Table 5:** XRD analysis data.

Compound	Pos. [2Th.]	Height [cts]	FWHM [2Th.]	d-Spacing [Å]	Rel. Int. [%]
Zn (II)	22.7560	169.85	0.1279	3.90457	100.00
Cu (II)	31.8381	32.61	0.1092	2.80845	100.00
Fe (III)	19.767	40.99	0.1535	4.48754	100.00
Se (IV)	19.7678	40.99	0.1535	2.80542	100.00

**Table 6:** Antioxidant enzyme activities in groups treated with either ceftriaxone or ceftriaxone metal complexes.

Group Items	Control	CFX	CFX/Mg	CFX/Zn	CFX/Se	CFX/Cu	CFX/Fe
ALT (U/L)	9.68 ± 1.02 ^g^	89.38 ± 4.69 ^a^	12.98 ± 1.98 ^f^	15.58 ± 2.36 ^e^	19.36 ± 2.56 ^d^	22.39 ± 1.69 ^c^	23.69 ± 2.02 ^bc^
AST (U/L)	29.36 ± 2.36 ^g^	203.36 ± 4.69 ^a^	50.69 ± 3.69 ^f^	55.98 ± 4.25 ^e^	72.25 ± 3.69 ^d^	79.58 ± 3.69 ^c^	82.35 ± 2.69 ^bc^
MDA (U/g)	9.68 ± 1.02 ^g^	123.65 ± 6.25 ^a^	18.52 ± 2.99 ^e^	16.52 ± 2.69 ^f^	24.28 ± 2.69 ^d^	29.68 ± 3.69 ^c^	31.02 ± 2.69 ^bc^
GSH (nmol/100 mg)	18.69 ± 1.69 ^a^	6.98 ± 0.98 ^g^	15.68 ± 2.05 ^bc^	14.69 ± 1.69 ^c^	12.97 ± 2.58 ^d^	9.01 ± 1.69 ^f^	10.39 ± 1.69 ^ef^
SOD (U/g)	13.69 ± 2.25 ^ab^	5.25 ± 0.35 ^e^	12.66 ± 1.69 ^b^	11.36 ± 1.58 ^c^	10.98 ± 1.69 ^d^	10.32 ± 1.69 ^d^	10.02 ± 0.69 ^d^
CAT (U/g)	15.69 ± 1.69 ^ab^	4.36 ± 0.98 ^f^	13.25 ± 1.65 ^b^	13.05 ± 1.69 ^b^	12.69 ± 2.68 ^c^	10.39 ± 2.69 ^e^	11.65 ± 1.69 ^d^

Values are means ± standard error. Mean values with different letters in the same row show significance at *p* ≤ 0.05, where the highest mean value has the symbol ^a^ and ^b−g^: Decreases in value were assigned alphabetically, with similar letters implying partial or complete non-significance and different letters implying significance. CFX = group supplemented with ceftriaxone; CFX/Mg = group supplemented with ceftriaxone/Mg complex; CFX/Zn = group supplemented with ceftriaxone/Zn complex; CFX/Se = group supplemented with ceftriaxone/Se complex; CFX/Cu = group supplemented with ceftriaxone/Cu complex.

**Table 7:** Inhibition zone diameter (mm/mg sample) of CFX and CFX metal complexes.

Sample	Inhibition Zone Diameter (mm/mg Sample)				
	*Bacillus subtilis* (G^+^)	*Streptococcus pneumoniae* (G^+^)	*Staphylococcus aureus* (G^+^)	*Escherichia coli* (G^−^)	*Pseudomonas aeruginosa* (G^−^)
Control (DMSO)	0.0 ± 0.0 ^c^	0.0 ± 0.0 ^d^	0.0 ± 0.0 ^e^	0.0 ± 0.0 ^d^	0.0 ± 0.0 ^d^
Ceftriaxone (CFX)	3.80 ± 0.11 ^b^	5.8 ± 0.73 ^c^	6± 0.89 ^d^	3.76 ± 0.31 ^c^	2.89 ± 0.45 ^c^
Zn (II)–CFX	12 ± 0.62 ^a^	16 ± 0.21 ^a^	12 ± 0.58 ^b^	10.98 ± 0.96 ^a^	8.90 ± 0.85 ^a^
Se (III)–CFX	11 ± 0.64 ^a^	9.50 ± 0.91 ^b^	17 ± 0.37 ^c^	15.98 ± 0.85 ^a^	12.76 ± 0.59 ^a^

Means within the same column (mean ± SE) carrying different letters are significant at *p* ≤ 0.05 using Duncan’s multiple range test, where the highest mean value has the symbol ^a^ and ^b−e^ those decreasing in value are assigned alphabetically.

## Data Availability

All the data are available inside the text.
